# Normal early development in siblings with novel compound heterozygous variants in *ASPM*

**DOI:** 10.1038/s41439-019-0088-0

**Published:** 2020-01-06

**Authors:** Taro Moriwaki, Narutoshi Yamazaki, Tetsumin So, Motomichi Kosuga, Osamu Miyazaki, Yoko Narumi-Kishimoto, Tadashi Kaname, Gen Nishimura, Torayuki Okuyama, Yasuyuki Fukuhara

**Affiliations:** 1 Division of Medical Genetics, National Center for Child Health and Development, 2-10-1 Okura, Setagaya-ku, Tokyo 157-8535 Japan; 2 Department of Clinical Laboratory Medicine, National Center for Child Health and Development, 2-10-1 Okura, Setagaya-ku, Tokyo 157-8535 Japan; 3 Division of Critical Care Medicine, National Center for Child Health and Development, 2-10-1 Okura, Setagaya-ku, Tokyo 157-8535 Japan; 4 Department of Radiology, National Center for Child Health and Development, 2-10-1 Okura, Setagaya-ku, Tokyo 157-8535 Japan; 5 Medical Genome Center, National Center for Child Health and Development, 2-10-1 Okura, Setagaya-ku, Tokyo 157-8535 Japan; 6 Department of Genome Medicine, National Center for Child Health and Development, 2-10-1 Okura, Setagaya-ku, Tokyo 157-8535 Japan; 7 Center of Intractable Diseases, Saitama Medical University Hospital, Saitama, Japan

**Keywords:** Disease genetics, Diseases

## Abstract

Autosomal recessive primary microcephaly 5 (MCPH5) is caused by pathogenic variants in *ASPM*. Using whole-exome sequencing, we diagnosed two siblings with MCPH5. A known pathogenic variant (NM_018136.4: c.9697C > T, p.(Arg3233*)) and a novel pathogenic variant (c.1402_1406del, p.(Asn468Serfs*2)) of *ASPM* were identified in affected siblings with normal intelligence. Their pathogenic variants were not located in the critical regions of *ASPM*, but the relationship between the genotypes and their normal intelligence was unclear.

Microcephaly is an anthropological/descriptive sign rather than a specific diagnosis and is used to denote a significant reduction in the occipital–frontal circumference (OFC) when compared with the age-matched peer group^1^. Based on the etiology, microencephaly can be classified as primary (genetic) or secondary (nongenetic or environmental)^2^. Primary microcephaly is characterized by the prenatal onset of abnormal brain growth, resulting in a reduced brain volume (OFC ≥ 2 standard deviations (SDs)^3^ or 3 SDs^4^ below the age- and sex-matched means at birth). Most cases of primary microcephaly show an autosomal recessive mode of inheritance, and 18 microcephaly primary hereditary (MCPH; MIM #251200) loci have been mapped to date (termed MCPH1–MCPH18)^5^. These genes affect not only cell cycle regulation but also DNA repair^6,7^. MCPH5 (MIM #605481) is the most frequently reported type, accounting for up to 40% of cases in both consanguineous and nonconsanguineous families^8^. Microcephaly patients may have mild-to-severe mental retardation, short stature, seizures, or hereditary hearing loss^9^. The phenotypes among MCPH1–MCPH18 are quite similar; therefore, exome sequencing is required for a precise diagnosis. Most patients with MCPH5 have been reported from the Middle East, and in East Asia, MCPH5 is rare, with just one sporadic case reported from Japan^3^. We herein report normal early development in a pair of Korean siblings with MCPH5 who had compound heterozygous *ASPM* (MIM#605481) pathogenic variants that included a novel deletion.

The primary patient was a 3-year-old girl who had been born in Korea as the first live-born child of nonconsanguineous Korean parents. She had no familial history of microcephaly. She was suspected of having agenesis of the corpus callosum on fetal ultrasound. She was born at 39 weeks and 6 days of gestation. Her birth weight was 2940 g (− 0.2SD), her length was 47 cm (− 0.7SD), and her OFC was 30 cm (− 2.0SD). She was referred to our hospital at 10 months because her parents moved to Japan for business. She had microcephaly and craniofacial dysmorphic features, including trigonocephaly and a small, sloped forehead. Her anterior fontanelle had already closed. Brain magnetic resonance imaging (MRI) and computed tomography (CT) revealed metopic suture craniosynostosis, which caused her trigonocephaly. Her brain had a simplified gyral pattern. In addition, significant small frontal lobes, small pons, and enlarged lateral ventricles were observed. Cerebellar hypoplasia was not observed. There was also a small arachnoid cyst in her left middle cranial fossa (Fig. 1). Developmental delay had not been noted since her infantile period. She held her head up steadily at 3 months, rolled over at 5 months, crawled at 11 months and walked alone at 1 year of age. At 10 months old, the Japanese Kyodaishiki developmental schedule showed that her intelligence quotient was 93 (motor func tion: 98, cognitive function: 89, verbal function: 89). She is now 3 years old, and her body weight is 12.2 kg (− 0.8 SD), her height is 89.5 cm (− 1.2 SD), and her OFC is 40.5 cm (− 4.7 SD). Standard karyotyping showed 46,XX, and an array comparative genomic hybridization chromosomal test revealed no pathogenic deletions or duplications. We performed next-generation sequencing to determine the etiology of her microcephaly with informed consent from her parents. Genomic DNA was extracted from the peripheral blood of the patient and from her parents. The ethics committees of the National Center for Child Health and Development approved this study.

**Fig. 1:**
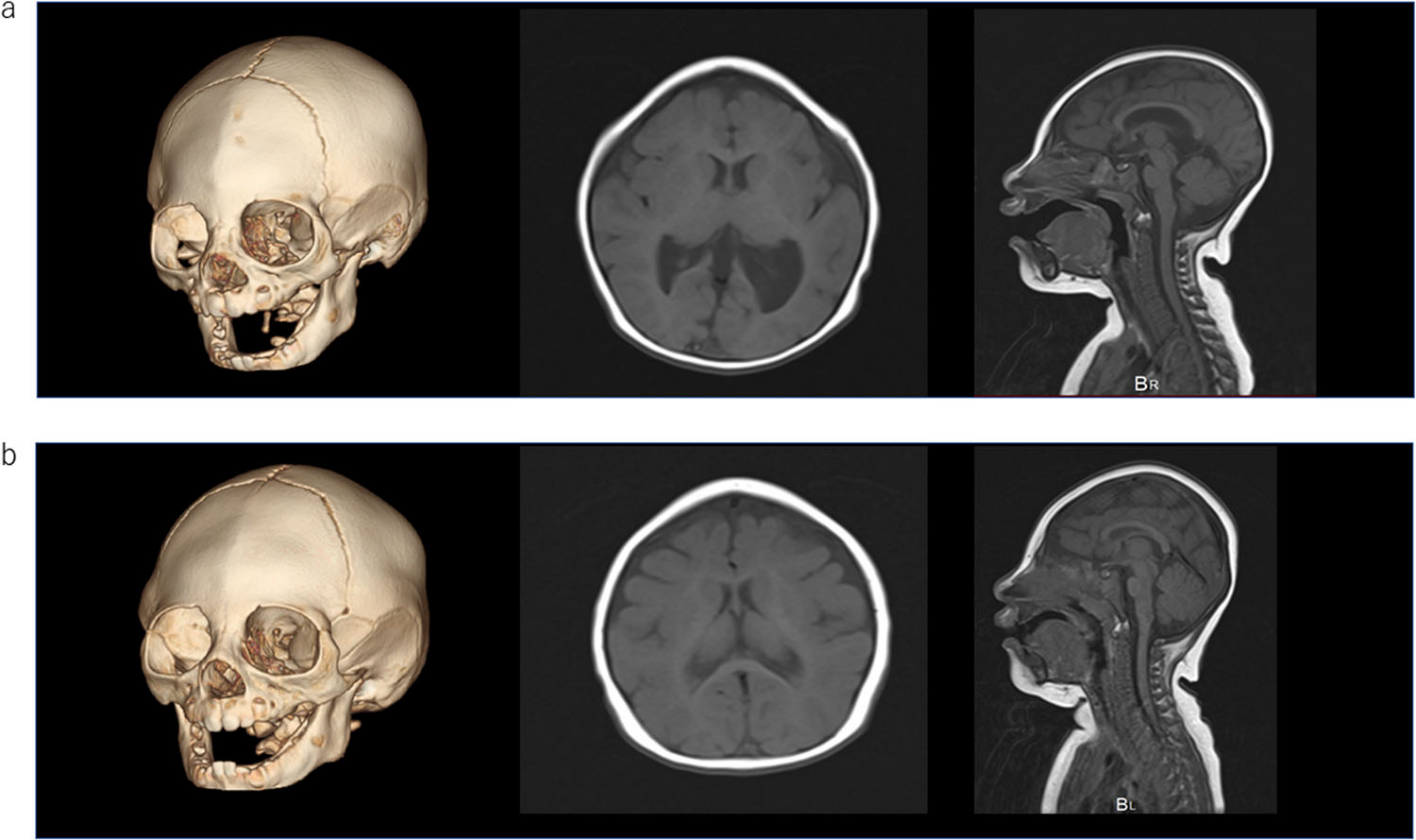
Brain imaging. **a** The older sister at 2 years of age. Three-dimensional CT (left), axial (middle), and sagittal (right) T1-weighted MRI. Metopic suture craniosynostosis led to trigonocephaly. Significant small frontal lobes, small pons, and enlarged lateral ventricles were observed. Cerebellar hypoplasia was not observed. **b** The younger brother at 6 months of age. The findings were similar to his sister’s, but he did not have lateral ventricle enlargement. Cerebellar hypoplasia was not observed, just as in his sister.

Her mother became pregnant while the genetic analysis for the primary patient was being performed. Fetal ultrasound revealed that the fetus was a male, and he also had microcephaly. The male patient was born at 38 weeks of gestation in Korea after his mother returned to her hometown. His birth weight was 2710 g (− 0.7 SD), his length was 45.5 cm (− 1.7 SD), and his OFC was 31.5 cm (− 1.3 SD). He was admitted to the neonatal intensive-care unit because he had microcephaly and transient tachypnea of the newborn (TTN). However, he recovered from the TTN quickly and finished all of his checkups, and he was able to be discharged at 10 days old. The brain MRI and CT findings were similar to his sister’s findings and suggested microcephaly. Metopic suture craniosynostosis, a simplified gyral pattern and small frontal lobes were noted. He also had a small pons but no lateral ventricle enlargement. Cerebellar hypoplasia was not observed, just as in his sister (Fig. 1). At 3 months old, his body weight was 6.93 kg (+ 0.7 SD), his height was 59.8 cm (− 0.7 SD), and his OFC was 35 cm (− 2.9 SD). At 7 months old, his intelligence quotient was 102 (motor function: 93, cognitive function: 109, verbal function: 97). His sister’s pathogenic variants of *ASPM* had already been detected; therefore, his DNA was also analyzed using Sanger sequencing (Fig. 2).

**Fig. 2:**
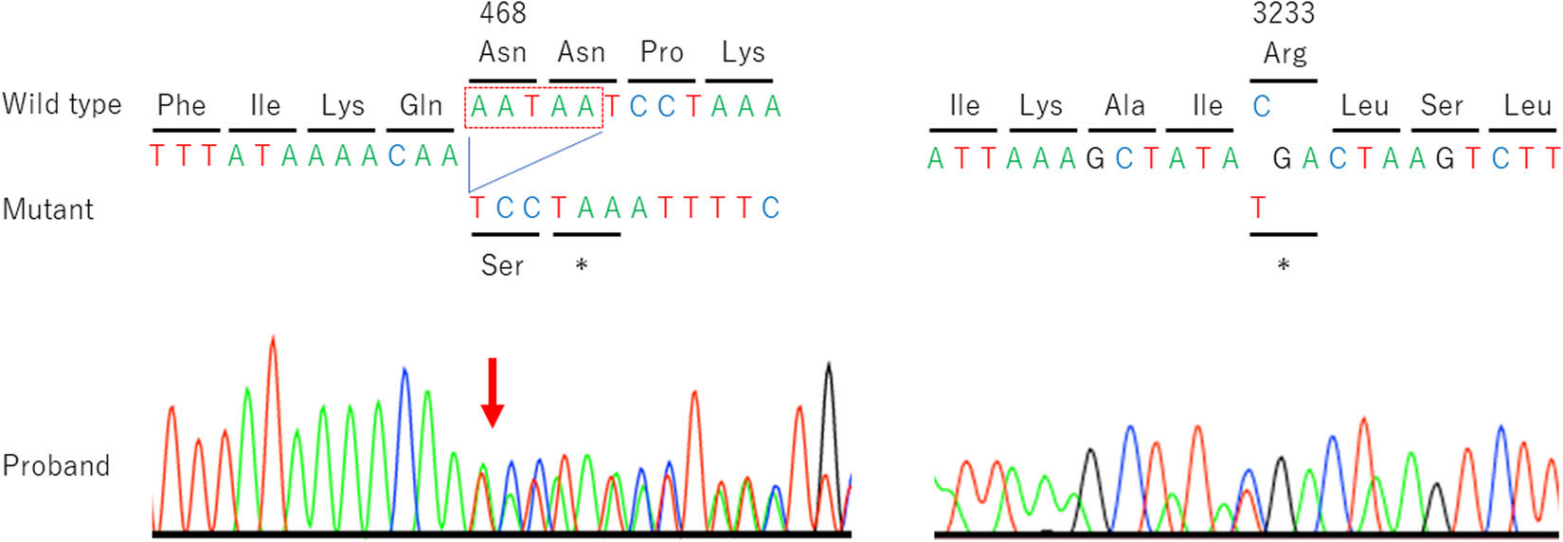
Partial sequence electropherograms of *ASPM* in the proband. The sequences around codons 468 and 3233 are shown. The sister was analyzed by whole-exome sequencing, and the brother was analyzed only by Sanger sequencing. The red box indicates the deleted sequence.

Regarding the methods, genomic DNA was extracted from the peripheral blood using a QIAamp DNA Mini kit (Qiagen GmbH, Hilden, Germany). Whole-exome sequencing and analyses were performed as described previously^10^. In brief, the SureSelect Human All Exon V6 kit (Agilent Technology, Santa Clara, CA, USA) was used for exon capture, and a HiSeq2500 system (Illumina, San Diego, CA, USA) with 101-bp paired-end reads was used for sequencing. Reads were aligned to GRCh37 using the Burrows-Wheeler Aligner (http://biobwa.sourceforge.net/). Variants were called using the GATK Unified Genotyper and ANNOVAR (http://annover.openbioinfomatics.org/en/latest/). Detected *ASPM* variants were validated by Sanger sequencing.

These analyses detected two different truncating pathogenic variants of *ASPM* in the sister first and then brother, NM_018136.4: c.1402_1406del, p.(Asn468Serfs*2) and c.9697C > T, p.(Arg3233*). Her father was a heterogeneous carrier of c.1402_1406del, p.(Asn468Serfs*2), and her mother was a carrier of c.9697C > T, p.(Arg3233*), suggesting that the children had these pathogenic variants in a compound heterozygous state. The variant c.1402_1406del, p.(Asn468Serfs*2) was novel and not registered in various databases, such as HGVD, 1000 Genomes data, ExAC, gnomAD, and iJGVD. Mutation Taster, a prediction program, estimated the variant to be disease causing. In addition, the variant was classified as pathogenic according to ACMG guidelines. The variant c.9697C > T, p.(Arg3233*) was recurrent in MCPH5 patients. It was also classified as pathogenic according to ACMG guidelines. Both variants were estimated to result in mRNA decay because both premature stop codons are further than 50 nucleotides upstream of the last exon–exon junction.

The siblings have craniosynostosis of metopic suture and trigonocephaly in common, and these factors are associated with their similar facial appearance. As in previous reports^11^, they also had a simplified gyral pattern, small frontal lobes, and small pons. The sister had ventricular enlargement, but not the brother. Neither had a small cerebellar vermis. The sister’s arachnoid cyst in the left middle cranial fossa was thought to have developed accidentally. MCPH5 patients usually have mild-to-severe intellectual disability^11^. However, developmental tests revealed the daughter’s intelligence quotient to be 93 (motor function: 98, cognitive function: 89, verbal function: 89) at 10 months old and the son’s intelligence quotient to be 102 (motor function: 93, cognitive function: 109, verbal function: 97) at 7 months old, showing no developmental delay, at least during early infancy. Previously, a patient with normal motor development was reported^12^. Language development is thought to start being delayed when patients are older than 3 years of age^13^. Of note, most patients have been reported to have hyperactivity and/or speech problems^11^; therefore, we need to keep monitoring these siblings to determine whether they properly acquire the ability to learn and use language with age. *ASPM* pathogenic variants were reported to spread throughout the coding sequence of the gene, with no obvious genotype–phenotype correlation^8^. The ASPM protein forms a complex with another protein linked to microcephaly: the microtubule-severing ATPase katanin. The ASPM/katanin complex controls microtubule disassembly at spindle poles, and its misregulation can lead to microcephaly^12^. The ASPM protein contains an N-terminal major sperm protein domain, a long unstructured region, calponin homology domains that can bind microtubules, multiple isoleucine–glutamine (IQ) motifs and a C-terminal HEAT repeat region^12^. The katanin p60N/p80C heterodimer interacts with the ASPM protein through its unstructured region, which contains several conserved peptide repeats^14^. The pathogenic variants (c.1402_1406del, p.(Asn468Serfs*2) and c.9697C > T, p.(Arg3233*)) are located in the IQ motifs and C-terminal HEAT repeat region, respectively, which may explain why our patients had normal intelligence. However, Okamoto et al.^3^ reported a Japanese patient who had *ASPM* pathogenic variants in exons 18 and 24 and showed severe intellectual disability. In addition, Mohamed’s 21 Egyptian patients had a high proportion of consanguinity and consequently included a high percentage of patients with homozygous *ASPM* pathogenic variants^11^. Notably, the patients with homozygous variants in exon 3, where the CH domains are located, showed severe intellectual disability, and those with homozygous variants in exons 17, 18, 21, and 23, where the functional importance is unclear, also showed severe intellectual disability^11^. Furthermore, even among the pedigree of exon 17, intellectual disability among patients ranged from severe to mild^11^. Given these findings, it is difficult to wholly attribute the normal intelligence of our patients to their genotype.

## Data Availability

The relevant data from this Data Report are hosted at the Human Genome Variation Database at 10.6084/m9.figshare.hgv.2801
